# 5-Imino-3,4-diphenyl-1*H*-pyrrol-2-one

**DOI:** 10.1107/S1600536814001032

**Published:** 2014-01-18

**Authors:** Evgeny Bulatov, Tatiana Chulkova, Matti Haukka

**Affiliations:** a Department of Chemistry, Saint Petersburg State University, Universitetsky Pr. 26, 198504 Stary Petergof, Russian Federation; b Department of Chemistry, University of Jyvaskyla, PO Box 35 FI-40014 Jyväskylä, Finland

## Abstract

The title compound, C_16_H_12_N_2_O, exists in the crystalline state as the 5-imino-3,4-di­phenyl­-1*H*-pyrrol-2-one tautomer. The dihedral angles between the pyrrole and phenyl rings are 35.3 (2) and 55.3 (2)°. In the crystal, inversion dimers linked by pairs of N—H⋯N hydrogen bonds generate a graph-set motif of *R*_2_^2^(8) *via* N—H⋯N hydrogen bonds.

## Related literature   

For general background to 5-imino­pyrrol-2-ones, see: Alves *et al.* (2009 ▶). For crystal structures of related compounds, see: Zhang *et al.* (2004 ▶).
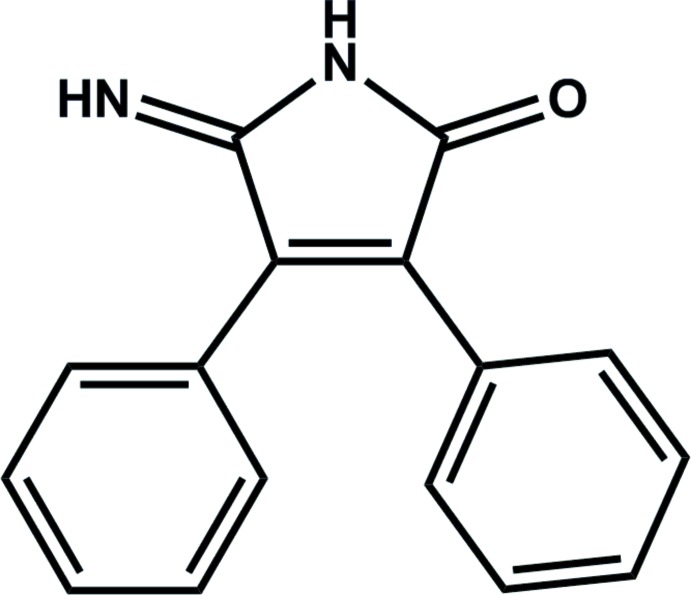


## Experimental   

### 

#### Crystal data  

C_16_H_12_N_2_O
*M* *_r_* = 248.28
Monoclinic, 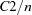

*a* = 19.687 (3) Å
*b* = 6.3064 (10) Å
*c* = 20.611 (3) Å
β = 97.850 (3)°
*V* = 2534.8 (7) Å^3^
*Z* = 8
Mo *K*α radiation
μ = 0.08 mm^−1^
*T* = 100 K
0.12 × 0.10 × 0.07 mm

#### Data collection  

Bruker KappaAPEXII diffractometer
Absorption correction: multi-scan (*SADABS*; Sheldrick, 2008*b* ▶) *T*_min_ = 0.990, *T*_max_ = 0.994
8351 measured reflections
2178 independent reflections
1360 reflections with *I* > 2σ(*I*)
*R*_int_ = 0.050

#### Refinement  

*R*[*F* ^2^ > 2σ(*F* ^2^)] = 0.043
*wR*(*F* ^2^) = 0.097
*S* = 1.00
2178 reflections
180 parameters
H atoms treated by a mixture of independent and constrained refinement
Δρ_max_ = 0.17 e Å^−3^
Δρ_min_ = −0.18 e Å^−3^

### 

Data collection: *APEX2* (Bruker, 2010 ▶); cell refinement: *APEX2*; data reduction: *APEX2*; program(s) used to solve structure: *SHELXS97* (Sheldrick, 2008*a* ▶); program(s) used to refine structure: *SHELXL97* (Sheldrick, 2008*a* ▶) and *SHELXLE* (Hübschle *et al.*, 2011 ▶); molecular graphics: *OLEX2* (Dolomanov *et al.*, 2009 ▶); software used to prepare material for publication: *SHELXL97*.

## Supplementary Material

Crystal structure: contains datablock(s) I. DOI: 10.1107/S1600536814001032/jj2181sup1.cif

Structure factors: contains datablock(s) I. DOI: 10.1107/S1600536814001032/jj2181Isup2.hkl

Supporting information file. DOI: 10.1107/S1600536814001032/jj2181Isup3.cml

CCDC reference: 978372

Additional supporting information:  crystallographic information; 3D view; checkCIF report

## Figures and Tables

**Table 1:** Hydrogen-bond geometry (Å, °)

*D*—H⋯*A*	*D*—H	H⋯*A*	*D*⋯*A*	*D*—H⋯*A*
N1—H1⋯N2^i^	0.93 (2)	1.96 (2)	2.882 (3)	172 (2)

Symmetry code: (i) 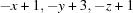.

**Fig. 1.**
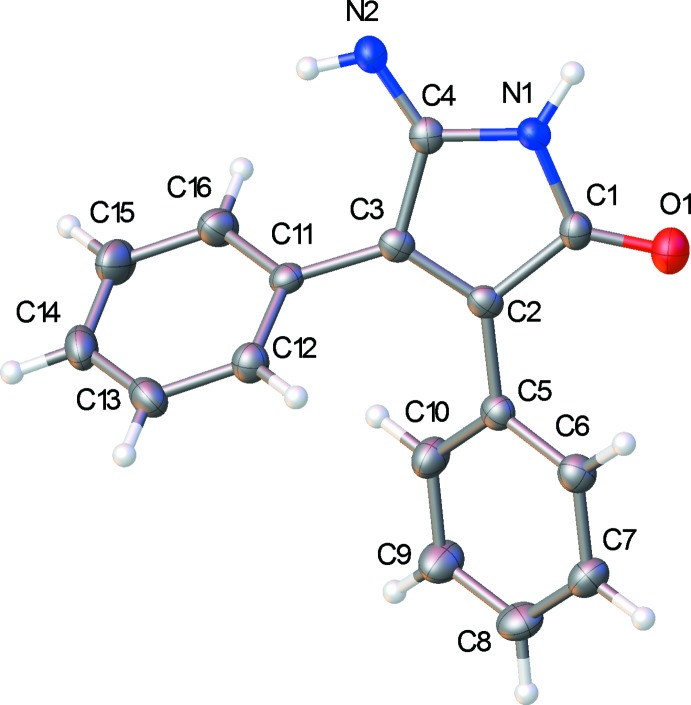
The molecular structure of the title compound with displacement ellipsoids drawn at the 50% probability level.

**Fig. 2.**
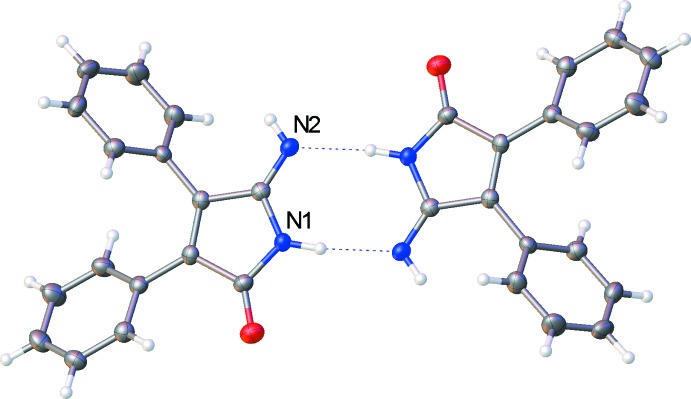
The structure of the R22(8) dimeric graph-set motif of the title compound.

## supplementary crystallographic information

### 1. Comment

The goal of this work was to determine which of the possible tautomers, *viz.* 5-Imino-3,4-di­phenyl-1*H*-pyrrol-2-one or 5-amino-3,4-di­phenyl-2H-pyrrol-2-one, is stabilized in the solid state.

In the title compound, the C1–N1 and C4–N1 bonds have the same length (1.380 (3) Å), which is longer than the C4–N2 bond length (1.271 (2) Å). In combination with the features of the difference Fourier map, this allows the unambiguous location of the hydrogen atom at the N1 atom. Thus, the title compound exists as 5-Imino-3,4-di­phenyl-1*H*-pyrrol-2-one in the crystalline state. Two monomeric title compounds are linked together by hydrogen bonds N–H•••N making a graph-set motif of *R*^2^_2_(8) (Table 1, Fig. 2).

### 2. Experimental

3,4-Di­phenyl-1*H*-pyrrol-2,5-di­imine (0.121 mmol, 0.030 g) was hydrolyzed in undried chloro­form (1 mL) for 1 week at room temperature. The yellow crystals of 5-Imino-3,4-di­phenyl-1*H*-pyrrol-2-one were obtained from the reaction mixture.

### 3. Refinement

The crystal of the title compound was immersed in cryo-oil, mounted in a Nylon loop, and measured at a temperature of 100 K. The X-ray diffraction data was collected on a Bruker Kappa Apex II diffractometer using MoKα radiation (λ = 0.71073 Å). The *APEX2* (Bruker AXS, 2010) program package was used for cell refinements and data reductions. The structure was solved by direct methods using the *SHELXS-97* (Sheldrick, 2008*a*) program. A multi-scan absorption correction based on equivalent reflections (*SADABS*, Sheldrick, 2008*b*) was applied to the data. Structural refinement was carried out using *SHELXL-97* (Sheldrick, 2008*a*) with the *Olex2* (Dolomanov *et al.*, 2009) and *SHELXLE* (Hübschle *et al.*, 2011) graphical user inter­faces.

The NH hydrogen atoms were located from a difference Fourier map and refined isotropically. Other hydrogen atoms were positioned geometrically and were also constrained to ride on their parent atoms, with C–H = 0.95 Å and U_iso_ = 1.2 U_eq_ (parent atom). The highest peak is located 1.08 Å from atom H6 and the deepest hole is located 0.98 Å from atom N1.

### Crystal data

**Table:**

C_16_H_12_N_2_O	*F*(000) = 1040
*M**_r_* = 248.28	*D*_x_ = 1.301 Mg m^−^^3^
Monoclinic, *C*2/*n*	Mo *K*α radiation, λ = 0.71073 Å
Hall symbol: -C 2ybc	Cell parameters from 1492 reflections
*a* = 19.687 (3) Å	θ = 3.1–22.6°
*b* = 6.3064 (10) Å	µ = 0.08 mm^−^^1^
*c* = 20.611 (3) Å	*T* = 100 K
β = 97.850 (3)°	Plate, yellow
*V* = 2534.8 (7) Å^3^	0.12 × 0.10 × 0.07 mm
*Z* = 8	

### Data collection

**Table:**

Bruker KappaAPEXII diffractometer	2178 independent reflections
Radiation source: fine-focus sealed tube	1360 reflections with *I* > 2σ(*I*)
Horizontally mounted graphite crystal monochromator	*R*_int_ = 0.050
Detector resolution: 9 pixels mm^-1^	θ_max_ = 25.1°, θ_min_ = 2.0°
φ scans and ω scans with κ offset	*h* = −23→20
Absorption correction: multi-scan (*SADABS*; Sheldrick, 2008*b*)	*k* = −7→7
*T*_min_ = 0.990, *T*_max_ = 0.994	*l* = −24→24
8351 measured reflections	

### Refinement

**Table:**

Refinement on *F*^2^	Primary atom site location: structure-invariant direct methods
Least-squares matrix: full	Secondary atom site location: difference Fourier map
*R*[*F*^2^ > 2σ(*F*^2^)] = 0.043	Hydrogen site location: inferred from neighbouring sites
*wR*(*F*^2^) = 0.097	H atoms treated by a mixture of independent and constrained refinement
*S* = 1.00	*w* = 1/[σ^2^(*F*_o_^2^) + (0.045*P*)^2^ + 0.1005*P*] where *P* = (*F*_o_^2^ + 2*F*_c_^2^)/3
2178 reflections	(Δ/σ)_max_ < 0.001
180 parameters	Δρ_max_ = 0.17 e Å^−^^3^
0 restraints	Δρ_min_ = −0.18 e Å^−^^3^

### Special details

**Table:**

Geometry. All e.s.d.'s (except the e.s.d. in the dihedral angle between two l.s. planes) are estimated using the full covariance matrix. The cell e.s.d.'s are taken into account individually in the estimation of e.s.d.'s in distances, angles and torsion angles; correlations between e.s.d.'s in cell parameters are only used when they are defined by crystal symmetry. An approximate (isotropic) treatment of cell e.s.d.'s is used for estimating e.s.d.'s involving l.s. planes.
Refinement. Refinement of *F*^2^ against ALL reflections. The weighted *R*-factor *wR* and goodness of fit *S* are based on *F*^2^, conventional *R*-factors *R* are based on *F*, with *F* set to zero for negative *F*^2^. The threshold expression of *F*^2^ > σ(*F*^2^) is used only for calculating *R*-factors(gt) *etc*. and is not relevant to the choice of reflections for refinement. *R*-factors based on *F*^2^ are statistically about twice as large as those based on *F*, and *R*- factors based on ALL data will be even larger.

### Fractional atomic coordinates and isotropic or equivalent isotropic displacement parameters (Å^2^)

**Table:**

	*x*	*y*	*z*	*U*_iso_*/*U*_eq_	
O1	0.57199 (8)	1.0248 (2)	0.40578 (7)	0.0350 (4)	
N1	0.50193 (9)	1.2453 (3)	0.45727 (8)	0.0223 (4)	
H1	0.5344 (12)	1.344 (4)	0.4747 (11)	0.051 (8)*	
N2	0.40778 (11)	1.4207 (3)	0.48842 (8)	0.0247 (5)	
H2	0.3658 (11)	1.411 (3)	0.4860 (10)	0.024 (7)*	
C1	0.51464 (11)	1.0785 (3)	0.41730 (9)	0.0222 (5)	
C2	0.44609 (10)	0.9837 (3)	0.39176 (9)	0.0205 (5)	
C3	0.39760 (10)	1.0984 (3)	0.41626 (9)	0.0202 (5)	
C4	0.43240 (11)	1.2715 (3)	0.45738 (9)	0.0208 (5)	
C5	0.43756 (10)	0.8056 (3)	0.34521 (9)	0.0219 (5)	
C6	0.48541 (11)	0.6423 (3)	0.34911 (10)	0.0257 (5)	
H6	0.5242	0.6466	0.3820	0.031*	
C7	0.47724 (11)	0.4730 (3)	0.30569 (10)	0.0290 (6)	
H7	0.5105	0.3631	0.3086	0.035*	
C8	0.42056 (12)	0.4650 (4)	0.25831 (10)	0.0331 (6)	
H8	0.4143	0.3477	0.2292	0.040*	
C9	0.37286 (12)	0.6268 (4)	0.25302 (10)	0.0358 (6)	
H9	0.3341	0.6215	0.2200	0.043*	
C10	0.38148 (11)	0.7966 (3)	0.29575 (10)	0.0289 (6)	
H10	0.3489	0.9086	0.2914	0.035*	
C11	0.32263 (10)	1.0668 (3)	0.40864 (9)	0.0203 (5)	
C12	0.29601 (11)	0.8701 (4)	0.42262 (9)	0.0266 (5)	
H12	0.3263	0.7584	0.4383	0.032*	
C13	0.22600 (11)	0.8354 (4)	0.41389 (10)	0.0310 (6)	
H13	0.2084	0.7004	0.4235	0.037*	
C14	0.18171 (11)	0.9967 (4)	0.39126 (10)	0.0337 (6)	
H14	0.1337	0.9719	0.3842	0.040*	
C15	0.20730 (12)	1.1938 (4)	0.37900 (11)	0.0381 (6)	
H15	0.1766	1.3061	0.3649	0.046*	
C16	0.27750 (11)	1.2298 (4)	0.38699 (10)	0.0303 (6)	
H16	0.2947	1.3656	0.3777	0.036*	

### Atomic displacement parameters (Å^2^)

**Table:**

	*U*^11^	*U*^22^	*U*^33^	*U*^12^	*U*^13^	*U*^23^
O1	0.0264 (10)	0.0386 (10)	0.0404 (9)	0.0027 (8)	0.0056 (7)	−0.0014 (8)
N1	0.0190 (11)	0.0212 (11)	0.0262 (10)	−0.0010 (9)	0.0010 (8)	−0.0029 (9)
N2	0.0183 (12)	0.0277 (12)	0.0279 (10)	−0.0014 (10)	0.0024 (9)	−0.0032 (9)
C1	0.0191 (13)	0.0234 (13)	0.0240 (11)	0.0016 (10)	0.0029 (10)	0.0030 (10)
C2	0.0218 (12)	0.0175 (12)	0.0214 (10)	−0.0006 (10)	0.0000 (9)	0.0029 (9)
C3	0.0224 (13)	0.0185 (12)	0.0192 (10)	0.0001 (10)	0.0005 (9)	0.0034 (9)
C4	0.0217 (13)	0.0224 (13)	0.0181 (11)	0.0010 (10)	0.0023 (9)	0.0039 (10)
C5	0.0219 (12)	0.0232 (13)	0.0212 (11)	−0.0012 (10)	0.0046 (10)	0.0008 (9)
C6	0.0254 (13)	0.0268 (13)	0.0242 (11)	0.0004 (11)	0.0006 (10)	0.0012 (10)
C7	0.0319 (14)	0.0263 (14)	0.0292 (11)	0.0058 (11)	0.0058 (11)	−0.0002 (10)
C8	0.0404 (15)	0.0331 (15)	0.0260 (12)	0.0016 (12)	0.0054 (12)	−0.0078 (11)
C9	0.0346 (15)	0.0417 (15)	0.0288 (12)	0.0064 (13)	−0.0043 (11)	−0.0087 (12)
C10	0.0267 (13)	0.0318 (14)	0.0278 (12)	0.0069 (11)	0.0017 (11)	−0.0029 (11)
C11	0.0225 (12)	0.0191 (13)	0.0193 (11)	0.0016 (10)	0.0026 (9)	−0.0031 (9)
C12	0.0249 (14)	0.0271 (14)	0.0278 (12)	0.0023 (11)	0.0034 (10)	0.0020 (10)
C13	0.0255 (14)	0.0345 (15)	0.0333 (13)	−0.0083 (12)	0.0045 (11)	−0.0052 (11)
C14	0.0186 (13)	0.0455 (17)	0.0366 (13)	−0.0004 (13)	0.0020 (10)	−0.0098 (12)
C15	0.0264 (15)	0.0378 (16)	0.0474 (15)	0.0089 (12)	−0.0050 (12)	−0.0044 (12)
C16	0.0277 (14)	0.0243 (14)	0.0373 (13)	0.0023 (11)	−0.0014 (11)	−0.0002 (11)

### Geometric parameters (Å, º)

**Table:**

O1—C1	1.233 (2)	C8—C9	1.381 (3)
N1—C4	1.379 (3)	C8—H8	0.9500
N1—C1	1.380 (3)	C9—C10	1.382 (3)
N1—H1	0.93 (2)	C9—H9	0.9500
N2—C4	1.271 (2)	C10—H10	0.9500
N2—H2	0.82 (2)	C11—C16	1.392 (3)
C1—C2	1.504 (3)	C11—C12	1.392 (3)
C2—C3	1.350 (3)	C12—C13	1.383 (3)
C2—C5	1.472 (3)	C12—H12	0.9500
C3—C11	1.476 (3)	C13—C14	1.378 (3)
C3—C4	1.490 (3)	C13—H13	0.9500
C5—C6	1.391 (3)	C14—C15	1.378 (3)
C5—C10	1.398 (3)	C14—H14	0.9500
C6—C7	1.388 (3)	C15—C16	1.388 (3)
C6—H6	0.9500	C15—H15	0.9500
C7—C8	1.380 (3)	C16—H16	0.9500
C7—H7	0.9500		
			
C4—N1—C1	110.67 (19)	C7—C8—H8	119.9
C4—N1—H1	123.5 (14)	C9—C8—H8	119.9
C1—N1—H1	124.7 (14)	C8—C9—C10	120.0 (2)
C4—N2—H2	111.4 (15)	C8—C9—H9	120.0
O1—C1—N1	124.8 (2)	C10—C9—H9	120.0
O1—C1—C2	128.67 (19)	C9—C10—C5	120.8 (2)
N1—C1—C2	106.56 (18)	C9—C10—H10	119.6
C3—C2—C5	129.01 (19)	C5—C10—H10	119.6
C3—C2—C1	107.61 (17)	C16—C11—C12	118.9 (2)
C5—C2—C1	123.28 (18)	C16—C11—C3	121.32 (19)
C2—C3—C11	129.49 (19)	C12—C11—C3	119.81 (19)
C2—C3—C4	108.16 (18)	C13—C12—C11	120.7 (2)
C11—C3—C4	122.32 (18)	C13—C12—H12	119.7
N2—C4—N1	122.4 (2)	C11—C12—H12	119.7
N2—C4—C3	130.6 (2)	C14—C13—C12	120.0 (2)
N1—C4—C3	106.92 (18)	C14—C13—H13	120.0
C6—C5—C10	118.24 (19)	C12—C13—H13	120.0
C6—C5—C2	120.76 (19)	C15—C14—C13	119.8 (2)
C10—C5—C2	120.99 (19)	C15—C14—H14	120.1
C7—C6—C5	120.96 (19)	C13—C14—H14	120.1
C7—C6—H6	119.5	C14—C15—C16	120.6 (2)
C5—C6—H6	119.5	C14—C15—H15	119.7
C8—C7—C6	119.7 (2)	C16—C15—H15	119.7
C8—C7—H7	120.1	C15—C16—C11	119.9 (2)
C6—C7—H7	120.1	C15—C16—H16	120.0
C7—C8—C9	120.3 (2)	C11—C16—H16	120.0

### Hydrogen-bond geometry (Å, º)

**Table:**

*D*—H···*A*	*D*—H	H···*A*	*D*···*A*	*D*—H···*A*
N1—H1···N2^i^	0.93 (2)	1.96 (2)	2.882 (3)	172 (2)

Symmetry code: (i) −*x*+1, −*y*+3, −*z*+1.
